# The Key Transcription Factor Expression in the Developing Vestibular and Auditory Sensory Organs: A Comprehensive Comparison of Spatial and Temporal Patterns

**DOI:** 10.1155/2018/7513258

**Published:** 2018-10-15

**Authors:** Shaofeng Liu, Yunfeng Wang, Yongtian Lu, Wen Li, Wenjing Liu, Jun Ma, Fuqin Sun, Mao Li, Zheng-Yi Chen, Kaiming Su, Wenyan Li

**Affiliations:** ^1^Department of Otolaryngology-Head and Neck Surgery, Yijisan Hospital of Wanan Medical College, Wuhu, Anhui 241001, China; ^2^ENT Institute and Otorhinolaryngology Department of Affiliated Eye and ENT Hospital, State Key Laboratory of Medical Neurobiology, Fudan University, Shanghai 200031, China; ^3^Key Laboratory of Hearing Medicine of NHFPC, Shanghai 200031, China; ^4^Otorhinolaryngology Department of the Shenzhen Second People's Hospital (First Affiliated Hospital of Shenzhen University), Shenzhen, China; ^5^Department of Otolaryngology and Program in Neuroscience, Harvard Medical School and Eaton Peabody Laboratory, Massachusetts Eye and Ear Infirmary, Boston, MA 02114, USA; ^6^Department of Otolaryngology, Head and Neck Surgery, Affiliated Sixth People's Hospital, Shanghai Jiao Tong University, Shanghai 200230, China

## Abstract

Inner ear formation requires that a series of cell fate decisions and morphogenetic events occur in a precise temporal and spatial pattern. Previous studies have shown that transcription factors, including Pax2, Sox2, and Prox1, play important roles during the inner ear development. However, the temporospatial expression patterns among these transcription factors are poorly understood. In the current study, we present a comprehensive description of the temporal and spatial expression profiles of Pax2, Sox2, and Prox1 during auditory and vestibular sensory organ development in mice. Using immunohistochemical analyses, we show that Sox2 and Pax2 are both expressed in the prosensory cells (the developing hair cells), but Sox2 is later restricted to only the supporting cells of the organ of Corti. In the vestibular sensory organ, however, the Pax2 expression is localized in hair cells at postnatal day 7, while Sox2 is still expressed in both the hair cells and supporting cells at that time. Prox1 was transiently expressed in the presumptive hair cells and developing supporting cells, and lower Prox1 expression was observed in the vestibular sensory organ compared to the organ of Corti. The different expression patterns of these transcription factors in the developing auditory and vestibular sensory organs suggest that they play different roles in the development of the sensory epithelia and might help to shape the respective sensory structures.

## 1. Introduction

The inner ear is a complex sensory organ responsible for both hearing and balance in vertebrates. Although the inner ear has an intricate structure and is multifunctional, its origin is quite simple, and both the hair cells and supporting cells of the inner ear arise from common progenitor cells [1, 2]. The formation of the inner ear requires that a series of cell fate decisions and morphogenetic events occur in a precise temporal and spatial pattern in order to subdivide these prosensory cells into their differentiated populations of hair cells and supporting cells [3, 4].

A number of different signaling pathways and transcription factors are known to be necessary for these developmental processes [5–8]. The paired-domain transcription factor Pax2, for example, plays a key role in regulating differential growth within the cochlear duct [9–12]. We also previously showed that Pax2 overexpression strongly promotes the proliferation of supporting cells in vitro [13]. The SRY-related HMG box (Sox) proteins are a group of transcription factors that regulate diverse developmental processes. A recent study demonstrated that Sox2, a member of the SoxB1 group along with Sox1 and Sox3, is required for the development of sensory epithelia, including the organ of Corti [14–17]. *Prox1* is the vertebrate homolog of the homeobox gene *prospero* in *Drosophila melanogaster*. Both *Prox1* and *prospero* play important roles in the development of various embryonic tissues and organs, such as the central nervous system [18, 19] and inner ear [7, 20, 21]. Thus, these three transcription factors are essential during inner ear development and sensory cell differentiation.

In previous study, we have already studied the gene expression in hair cells as well as surrounding cells of inner ear from E16 to P7 by a comprehensive cell type-specific RNA-Seq study [22], which provided a general idea about the expression tendency of Pax2, Sox2, and Prox1 in both hair cells and surrounding cells during the development of inner ear. While the spatial expression Pax2, Sox2, and Prox1 during inner ear development are also particularly important. Notably, the expression of Pax2 is one of the first indicators of otic placode induction, and it continues to be expressed in various regions of the ear throughout subsequent stages of development. In contrast, Sox2 is expressed in the neural tube and dorsolateral regions of murine otocysts at embryonic day 9.5 (E9.5), which is consistent with a role in sensory organ development. Prox1 is very weakly expressed in the most basal region of the developing mouse organ of Corti at E14.5. As the sensory epithelium differentiates, Prox1 becomes restricted to a subset of supporting cells, and this expression is consistent through the duration of embryogenesis and into the second postnatal week [20]. Although the expression patterns of Pax2, Sox2, and Prox1 have been investigated previously, there was no systematic study on the temporal and spatial expression profiles of these transcription factors during the development of cochlea, and the detailed expression pattern will help understand their functions.

In this study, we present a comprehensive description of the temporal and spatial expression profiles of Pax2, Sox2, and Prox1 during auditory and vestibular sensory organ development. Our results demonstrate that Pax2, Sox2, and Prox1 are differentially expressed and overlap in various regions of the developing inner ear. Comparison of their unique expression patterns facilitates our understanding on the individual underlying genes functions as well as the cochlear developmental process, meanwhile provided more clues for further investigating the relationships among those transcriptional factors on the sensory epithelium determination, progenitor cell proliferation, and hair cell differentiation during the inner ear development.

## 2. Results

### 2.1. The Expression Patterns of Pax2, Sox2, and Prox1 during the Sensory Epithelium Determination in Mouse Inner Ear (E9.5–E13.5)

**Figure 1:**
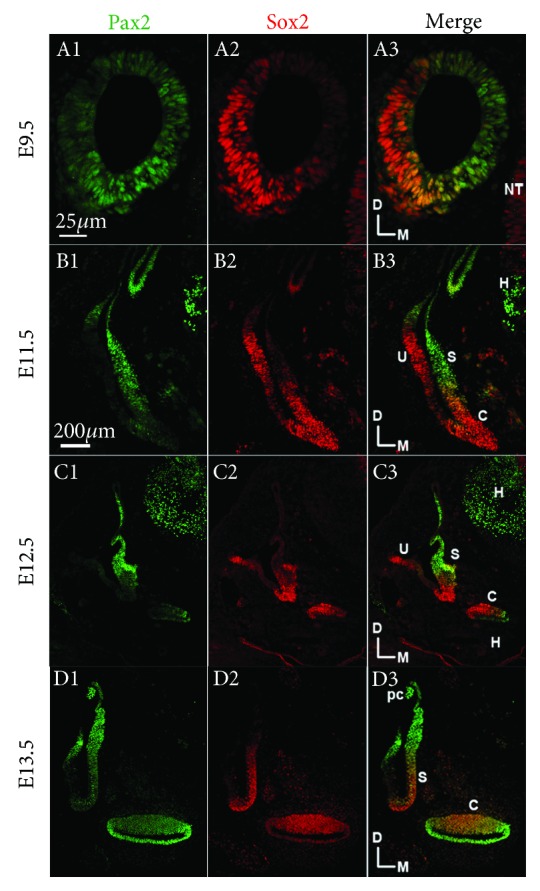
Comparison of Pax2, Sox2 expression in the inner ear from E9.5 to E13.5. Immunofluorescence staining of Sox2 (red), Pax2 (green), and Prox1 (green) in the mouse inner ear at E9.5, E11.5, E12.5, and E13.5. S: saccule; U: utricle; C: cochlea; D: dorsal; M: medial; pc: posterior crista; H: hindbrain; NT: neural tube. Scale bars = 25 *μ*m (A1–A3); 200 *μ*m (B1–B3, C1–C3, and D1–D3).

To better understand the role of Sox2 in mouse inner ear development, we first compared the pattern of Sox2 gene expression with that of Pax2 in specific presumptive sensory tissues by colabeling with antibodies against both proteins. Pax2 transcripts were previously shown to be initially distributed uniformly throughout the epithelium of the otic placode [23]. From E8.5 to E9.5, invagination of the otic placode continues and leads to the formation of the otic vesicle, and we found that during this period Pax2 distribution undergoes reorganization. At E9.5, Pax2 was expressed throughout the epithelium of the otic otocyst, but it was more concentrated in the centro-medial area next to the neural tube. In contrast, Sox2 was found in all sensory regions, with higher expression in the dorsolateral area of the otic vesicle (Figure 1, A1–A3).

The cochlear duct begins to develop as an out-pocketing of the otocyst at E11.5. And we found that two separate Sox2 expression domains were present at this time, a dorsal domain that corresponds to the sensory primordia of the utricle and a more ventral domain that corresponds to the sensory primordia of the saccule and the prosensory domain of the cochlea. Compared to Sox2, Pax2 expression only partially overlapped with the sensory regions of the saccule and the cochlea. Further, there was no obvious overlap of Pax2 expression with the macula of the utricle. At this stage, Pax2 expression was mostly restricted to the posterior region of the cochlear anlage (Figure 1, B1–B3).

Within the cochlear duct at E12.5, Sox2 was expressed in the medial half of the duct that appeared to correspond with the prosensory domain, specifically the population of cells that would give rise to the organ of Corti. This expression pattern partially overlapped with that of Pax2 but had obvious differences in distribution. Pax2 was mainly expressed in both sides of Reissner's membrane. Notably, Pax2 expression gradually weakened in the prosensory domain of the saccule at this developmental stage (Figure 1, C1–C3).

At E13.5, Pax2 was mainly located in the nonsensory domains of the vestibular organ and the cochlea. In contrast, Sox2 was gradually restricted to the prosensory domain, primarily in the medial half of the cochlear duct at the apex. A band of Sox2 expression was also observed within the basal region of the cochlear duct, which is correlated with the position of the developing organ of Corti and Kolliker's organ (Figure 1, D1–D3). At this stage, Prox1 expression was weakly detected in the sensory epithelia of the vestibular organ, but not in the cochlea (data not shown).

### 2.2. The Expression Patterns of Pax2, Sox2, and Prox1 during the Hair Cell Development in Mouse Inner Ear (E15.5-E18.5)

**Figure 2:**
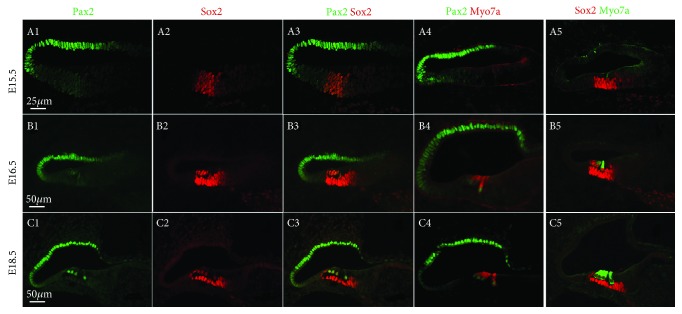
Comparison of Pax2, Sox2 expression in the cochlea. Immunofluorescence of Sox2, Pax2, and myosin7a expression in the cochlear duct at E15.5, E16.5, and E18.5. Scale bars = 25 *μ*m (A1–A5); 50 *μ*m (B1–B5 and C1–C5).

To relate Pax2, Sox2, and Prox1 expression to hair cell development, we colabeled the cells with the hair cell marker myosin7a. At E15.5, myosin7a was clearly expressed in developing inner hair cells, but not in the developing outer hair cells (OHCs) at the basal end of the cochlea (Figure 2, A4–A5). However, at E16.5, this expression expanded to include some of the OHCs (Figure 2, B4–B5), and at E18.5, expression was seen in all hair cells (Figure 2, C4–C5).

We observed an interesting phenomenon regarding Pax2 expression, whereby it was still expressed in Reissner's membrane and the stria vascularis, but it was not expressed in the hair cells when they began to express myosin7a at E15.5 (Figure 2, A1–A5). At later time points, Pax2 expression was upregulated in the hair cells of the organ of Corti and was stably expressed in these hair cells by E18.5 (Figure 2, B1–B5 and C1–C5).

At E15.5, Sox2 was still expressed in the medial half of the cochlear duct at the apex as well as within the basal region of the cochlear duct that is correlated with the position of the developing organ of Corti and Kolliker's organ (Figure 2, A1–A5). At later stages, Sox2 was expressed in both immature hair cells and supporting cells, and this expression was maintained in hair cells through E18.5 (Figure 2, B1–B5 and C1–C5).

**Figure 3:**
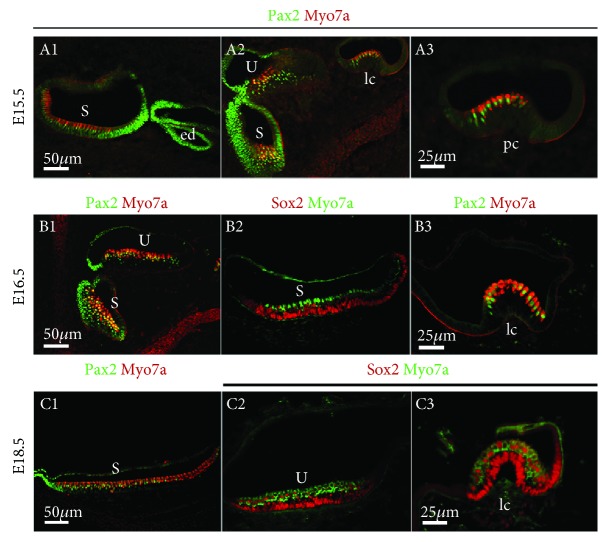
Comparison of Pax2 and Sox2 gene expression in the vestibule. Immunofluorescence of Pax2, Sox2, and myosin7a in the vestibule from E15.5 to 18.5. S: saccule; U: utricle; ed: endolymphatic duct; pc: posterior crista; lc: lateral crista. Scale bars = 50 *μ*m (A1–A2, B1–B2, and C1–C2); 25 *μ*m (A3, B3, and C3).

Expression of Pax2 in the utricle, saccule, and three cristae of the vestibular organ differed from the expression in the organ of Corti. At E16.5, Pax2 was still expressed in immature hair cells and supporting cells of the sensory epithelia of the saccule, but only in the hair cells of the crista ampullaris at E15.5 (Figure 3, A1–A3) and of the macula utriculi at E16.5 (Figure 3, B1–B3). After E18.5, Pax2 expression was restricted to the hair cells of the saccule (Figure 3, C1–C3).

**Figure 4:**
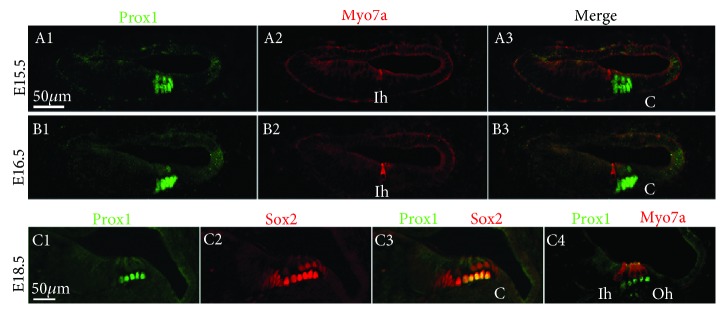
Prox1 gene expression patterns in the cochlea. Immunofluorescence of Prox1 (green) and myosin7a (red) in the cochlea at E15.5, E16.5, and E18.5. Scale bar = 25 *μ*m.

To determine the expression pattern of Prox1 during the hair cell differentiation of the inner ear, we colabeled embryonic sections using antibodies against Prox1 and Myo7a or Prox1 and Sox2. Prox1 was detected in the precursor cells in the most basal region of the developing cochlea until E15.5, including in the future outer hair cells (OHCs), Dieter's cells, and pillar cells (Figure 4, A1–A3 and B1–B3), consistent with an earlier description [20].

Prox1 induction also followed a base-to-apex gradient along the length of the cochlear duct, which is similar to Pax2 and Sox2. By E16.5, Prox1 was expressed in Dieters' cells and pillar cells of the basal region of the developing cochlea but could not be detected in the apical region. Kirjavainen et al. previously showed Prox1 expression in OHCs of the basal coil at postnatal day 0 (P0) [24]; however, we found that Prox1 was only transiently expressed in OHCs and was rapidly downregulated in OHCs while expression of Prox1 was sustained in the supporting cells through E18.5 (Figure 4, C1–C4). At this stage, the morphological distinction between the supporting cells and the hair cells was clear throughout the cochlea, and the supporting cells underlying the hair cells expressed high levels of Prox1. This was distinct from the Sox2 expression, which was found in all of the supporting cells and in a group of cells within Kolliker's organ (Figure 4, C1–C4). Notably, the inner phalangeal cells, border cells, and Hensen's cells did not express Prox1 at this stage (Figure 4, C1–C4). The supporting cells underlying the inner hair cells did not express Prox1, thus distinguishing these two adjacent populations of supporting cells.

**Figure 5:**
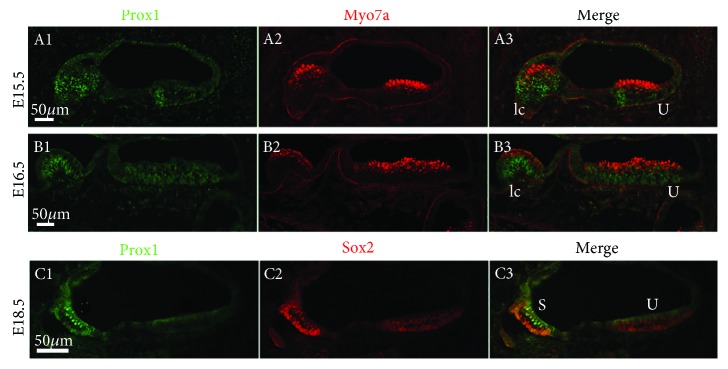
Prox1 expression in the prosensory domain of the developing vestibule. Immunofluorescence of Prox1 (green) and myosin7a/Sox2 (red) in the macula sacculi, macula utriculi, and crista ampullaris from E13.5 to E18.5. S: saccule; U: utricle; pc: posterior crista; lc: lateral crista. Scale bars = 50 *μ*m.

Prox1 was not detected in the utricle after E16.5 (Figure 5, A1–A3 and C1–C3). These data differ from an earlier study [24] in which the expression of Prox1 was maintained until P1, but they are consistent with the results of Bermingham-McDonogh et al. [20]. In contrast, Prox1 was still expressed in the saccule at E18.5 (Figure 5, A1–A3 and C1–C3).

### 2.3. The Expression Patterns of Pax2, Sox2, and Prox1 in Neonatal Mouse Inner Ear (P0-P7)

**Figure 6:**
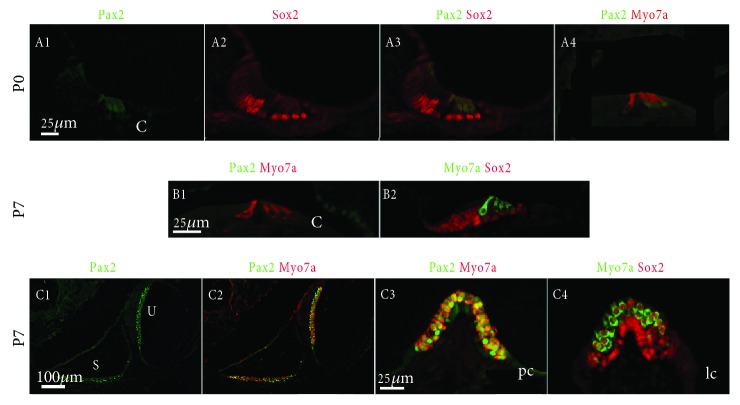
Pax2 and Sox2 gene expression patterns in the neonatal mouse inner ear (P0-P7). Scale bars = 25 *μ*m (A1–A4, B1–B2, and C3–C4); 100 *μ*m (C1–C2).

Pax2 expression was maintained in hair cells at P0, but the expression was notably weaker in the inner hair cells in the basal region of the cochlea (Figure 6, A1–A4). By P7, this protein was no longer detectable in any of the hair cells (Figure 6, B1–B2). Compared with Pax2, Sox2 expression was restricted to the supporting cells—including the inner phalangeal cells, inner pillar cells, outer pillar cells, Dieter's cells, and Hensen's cells—in the basal region of the cochlea by P0 as well as in a group of cells within Kolliker's organ (Figure 6, A1–A4). Furthermore, Pax2 was still expressed in the hair cells of the vestibular sensory epithelia at P7 (Figure 6, C1–C4), while Sox2 was found in both the hair cells and supporting cells (Figure 6, C1–C4).

**Figure 7:**
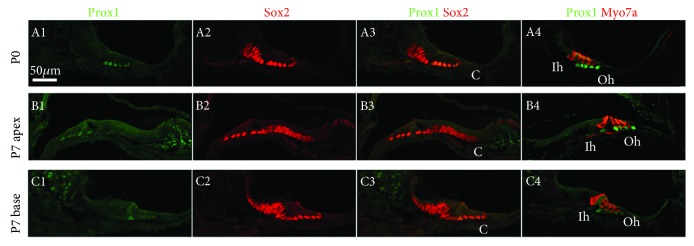
Prox1 is expressed in the neonatal mouse cochleae. Immunohistochemistry of Prox1, Sox2, and anti-myosin7a from P0 to P7. Scale bars = 50 *μ*m.

Prox1 expression continued in Dieters' cell and pillar cell nuclei after birth, but the intensity gradually weakened over the course of development (Figure 7, A1–A4). At P7, Prox1 immunoreactivity was noticeably reduced in subsets of supporting cells, particularly those in the first and second rows of Dieters' cells and in outer pillar cells in the apical region of the organ of Corti, and Prox1 was even more weakly expressed in the basal region (Figure 7, B1–B4 and C1–C4). This is similar to cProx1 expression in the basilar papilla in chicken where nuclear cProx1 expression is downregulated in most hair cells by stage 37 and in many supporting cells by stage 40 [25]. Prox1 was not detected in the saccule or ampulla after birth.

## 3. Discussion

### 3.1. Distinct Temporospatial Expression Patterns of Pax2 and Sox2 during the Development of Mouse Cochlea

It has been reported that Pax-2 is one of the earliest markers of the developing inner ear, which was expressed in the auditory and vestibular sensory primordia [26, 27] and related to the highly proliferative potential of progenitor cells in developing sensory patches of chicken [28]. Mutations in the Pax2 gene result in defects of the auditory and vestibular organs [9, 29]. Meanwhile, Sox2 is another earliest and critical gene for the development of the inner ear, which defined the prosensory domain during the development of the cochlea. The absence or reduced expression of Sox2 within the developing inner ear resulted the impaired development of sensory epithelium and followed hearing loss [15, 30].

One of the primary goals of this study was to determine the temporal and spatial relationships in the expression profiles of Pax2 and Sox2 in order to gain further insight into their possible functions. Our results demonstrate that Pax2 and Sox2 have a reciprocal relationship. In early development, they, respectively, define the medial and lateral sides of the otocyst, and as the sensory patches are innervated, Pax2 is downregulated in the prosensory domain. However, it is selectively upregulated in the hair cells when they begin to express myosin7a, and this expression is maintained in the cochlea until at least P0. This expression pattern suggests that Pax2 might have diverse roles in sensory cell development within the cochlea.

Sox2 expression in the mammalian inner ear initially correlates with the formation of prosensory domains before ultimately becoming restricted to the supporting cells [14–17]. The downregulation of Sox2 in the developing hair cells is required in order for a subset of these cells to differentiate into hair cells. While Sox2 has been shown to activate *Atoh1* by directly binding to consensus sequences in the *Atoh1* enhancer [14], Puligilla and Kelley found that Sox2 plays a dual role in inner ear formation [31]. Their work showed that Sox2 is initially required to specify prosensory competence, but the subsequent downregulation of Sox2 must occur in order to allow Atoh1 expression.

Using Pax2 as a marker for the prosensory cells, the initial overlap and subsequent differential expression of Sox2 suggests that these two proteins likely function in different molecular pathways that act to direct cells towards different cell fates. Thus, Sox2 might be used as a marker of prosensory cells/supporting cells in the cochlea.

### 3.2. Differential Expression of Sox2 and Prox1 during Cochlear Development

It has been reported that the Prox1 expressed during the development of vertebrate inner ear [25]. In the current study, we characterize the temporal and spatial expression of the mouse developing organ of Corti as well as the vestibular organs, as compared with the distribution of Sox2 and Myo7a. As shown, Sox2 is expressed in prosensory cells and subsequently in immature hair cells and the developing supporting cells before ultimately becoming restricted only to the supporting cells in the auditory epithelia, which suggests that this protein is involved in hair cell differentiation. Notably, the duration of Prox1 expression was shorter than that of Sox2, as it began later in development and was only transiently expressed in the presumptive hair cells and developing supporting cells. Furthermore, Prox1 was only expressed in a subset of developing supporting cells, and not in any mature supporting cells, suggesting that Prox1 might play different roles in supporting cell development depending on the stage of embryonic development. Prox1 has also been proposed to be a marker for the developing supporting cells [21]. Unfortunately, we cannot speculate on the interactions between Prox1 and Sox2 because their expression patterns did not overlap significantly in the presumptive hair cells and developing supporting cells. In addition to being a downstream target of Sox2, Prox1 also upregulates its own expression, which likely plays a role in hair cell development. Similarly, using cotransfection of Prox1 and Atoh1 in cochlear prosensory cells, Prox1 was shown to inhibit Atoh1-induced expression of Myo6, a hair cell marker, in nonsensory cells [30].

### 3.3. Differential Roles of Pax2, Sox2, and Prox1 during Vestibular and Auditory Organ Development

We found that Pax2 was expressed in all of the immature auditory hair cells in the mouse, but its expression gradually decreased and had completely disappeared by P7. In contrast, Pax2 protein expression was still evident in hair cells of the vestibular organ at P7. Similarly, Sox2 was not only expressed in prosensory cells but also expressed in the supporting cells and nascent hair cells of both the auditory and vestibular organs at very early stages of development. At P7, however, Sox2 was only expressed in the supporting cells of the organ of Corti, but it was still present in both hair cells and supporting cells of the vestibular sensory organ. Prox1 was transiently expressed in both presumptive hair cells and developing supporting cells, but it had a much shorter time course of expression compared to the total time for the cochlea to fully develop and mature, and it had much lower expression intensity in the sensory epithelia of the vestibular organ compared to the auditory organ.

The different expression patterns of Pax2 and Sox2 in the vestibular versus the auditory organs suggest that the expression of these proteins might be associated with different requirements in vestibular hair cell specialization [9, 32]. Both auditory and vestibular hair cells have similar functions and appear morphologically similar, but they have clear differences in terms of detailed structures such as ion channels, cilium structures, and innervation [33, 34].

The data presented here suggest that Pax2, Sox2, and Prox1 expression might be involved in this cell fate choice, and the different expression patterns of these proteins in the developing auditory and vestibular sensory organs might help to shape each respective sensory structure.

## 4. Conclusions

**Figure 8:**
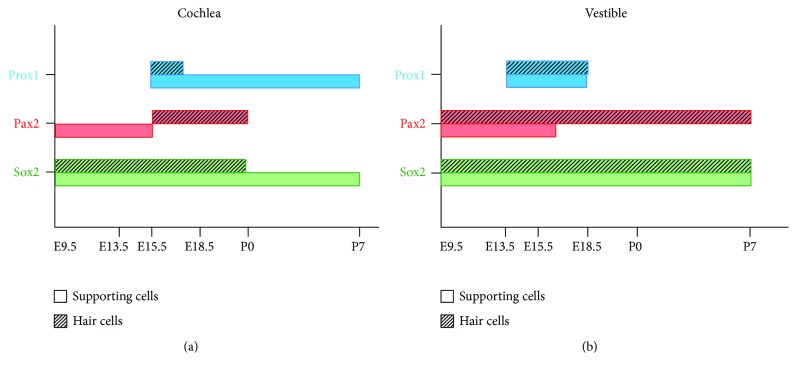
Schematic diagram of Sox2, Pax2, and Prox1 gene expression during mouse inner ear development.

Pax2, Sox2, and Prox1 have differential and overlapping expression patterns during auditory and vestibular sensory organ development. Sox2 and Pax2 are both expressed in the prosensory cell/developing hair cells, but Pax2 expression eventually disappear and Sox2 expression is subsequently restricted to the supporting cells of the organ of Corti. In contrast, in the vestibular organ, Pax2 is still clearly expressed in hair cells at P7, and Sox2 is still expressed in both hair cells and supporting cells. Prox1 is only transiently expressed in presumptive hair cells and developing supporting cells in the organ of Corti, and Prox1 has a weaker expression in the vestibular sensory organ compared to the auditory organ. The different expression patterns in the developing auditory and vestibular sensory organs suggest that Sox2 and Pax2 play different roles in the development of the sensory epithelia and might help to shape the corresponding sensory structures (Figure 8).

## 5. Materials and Methods

### 5.1. Animals

Pregnant C57/6 mice (Department of Laboratory Animal Science, Medical College of Fudan University) were time-mated and checked for plugs the following day to verify that they were pregnant. The plug date was designated as E0.5, and the day of birth was defined as P0. Pregnant mice were euthanized on E9.5, E11.5, E12.5, E13.5, E15.5, E16.5, and E18.5, according to the Guide of the Care and Use of Laboratory Animals. All animal procedures were performed according to the protocols approved by the Animal Care and Use Committee of Fudan University and were consistent with the National Institutes of Health Guide for the Care and Use of Laboratory Animals.

### 5.2. Cryosectioning

The embryonic mice were removed and decapitated, and the whole heads from E9.5 to E16.6 mice or otic bullae from E18.5 to P7 were fixed in 4% paraformaldehyde (Sigma-Aldrich) in 0.01 M phosphate-buffered saline (PBS, PH7.4) at 4°C. For P7 and older mice, decalcification was performed with 10% EDTA for 1-2 days at 4°C. Tissues were washed three times in phosphate-buffered saline (PBS), cryoprotected in successive changes of increasing sucrose concentrations (from 15% sucrose to 30% sucrose in PBS), and embedded in optimal cutting temperature compound (Sakura Finetek) at 4°C overnight. Sections with a thickness of 10 *μ*m were made with a Leica CM3050 cryostat (Leica).

### 5.3. Immunofluorescence

For immunofluorescence, tissue sections were washed in PBS and permeated with PBS/0.1% Triton X-100 for 40 min at 37°C, and subsequently incubated in a solution of 10% normal goat or donkey serum for 30 min at room temperature. After antigen retrieval in a 97.9°C water-bath with 10 mM Na-Citrate, pH 6.0, the samples were incubated overnight at 4°C with primary antibody. After incubation, samples were rinsed and incubated with an Alexa Fluor 488 donkey anti-goat or donkey anti-rabbit (1 : 200 dilution; Molecular Probes) for 1 h at 37°C. If double immunostaining was performed, Cy3-conjugated AffiniPure Donkey Anti-Goat IgG (H + L) (1 : 200 dilution; Jackson ImmunoResearch) was also used. The samples were then rinsed again, mounted with anti-fade medium (Slowfade Gold Antifade Reagent with DAPI, Molecular Probes), and observed with a Leica microscope. The following primary antibodies were used: Pax2 (1 : 200 dilution), Prox1 (1 : 1000 dilution), Sox2 (1 : 600 dilution) (all from Santa Cruz Biotechnology), and goat anti-myosin7a (1:200 dilution; Proteus Biosciences). Slides were analyzed by conventional fluorescence microscopy using a Nikon Eclipse TE2000-5 Fluorescence Microscope with a Nikon Digital Sight DS-U1 CCD camera. Images were acquired with the Nikon NIS-Elements D2.30 image manager, and Adobe Photoshop CS 2.0 was used to obtain the merged images and to adjust the contrast and brightness. All images were adjusted equally.

## Data Availability

The data used to support the findings of this study are available from the corresponding author upon request.

## References

[1] Fekete D. M., Muthukumar S., Karagogeos D. (1998). Hair cells and supporting cells share a common progenitor in the avian inner ear. *Journal of Neuroscience*.

[2] Xu J., Ueno H., Xu C. Y., Chen B., Weissman I. L., Xu P. X. (2017). Identification of mouse cochlear progenitors that develop hair and supporting cells in the organ of Corti. *Nature Communications*.

[3] Driver E. C., Northrop A., Kelley M. W. (2017). Cell migration, intercalation and growth regulate mammalian cochlear extension. *Development*.

[4] Fuchs J. C., Tucker A. S. (2015). Development and integration of the ear. *Current Topics in Developmental Biology*.

[5] Anwar M., Tambalo M., Ranganathan R., Grocott T., Streit A. (2017). A gene network regulated by FGF signalling during ear development. *Scientific Reports*.

[6] Hickox A. E., Wong A. C. Y., Pak K. (2017). Global analysis of protein expression of inner ear hair cells. *Journal of Neuroscience*.

[7] Nishimura K., Noda T., Dabdoub A. (2017). Dynamic expression of Sox2, Gata3, and Prox1 during primary auditory neuron development in the mammalian cochlea. *PLoS One*.

[8] Paxton C. N., Bleyl S. B., Chapman S. C., Schoenwolf G. C. (2010). Identification of differentially expressed genes in early inner ear development. *Gene Expression Patterns*.

[9] Bouchard M., de Caprona D., Busslinger M., Xu P., Fritzsch B. (2010). Pax2 and Pax8 cooperate in mouse inner ear morphogenesis and innervation. *BMC Developmental Biology*.

[10] Christophorou N. A. D., Mende M., Lleras-Forero L., Grocott T., Streit A. (2010). Pax2 coordinates epithelial morphogenesis and cell fate in the inner ear. *Developmental Biology*.

[11] Freter S., Muta Y., O'Neill P., Vassilev V. S., Kuraku S., Ladher R. K. (2012). Pax2 modulates proliferation during specification of the otic and epibranchial placodes. *Developmental Dynamics*.

[12] Pechriggl E. J., Bitsche M., Glueckert R. (2015). Development of the innervation of the human inner ear. *Developmental Neurobiology*.

[13] Chen Y., Yu H., Zhang Y. (2013). Cotransfection of Pax2 and Math1 promote in situ cochlear hair cell regeneration after neomycin insult. *Scientific Reports*.

[14] Kempfle J. S., Turban J. L., Edge A. S. B. (2016). Sox2 in the differentiation of cochlear progenitor cells. *Scientific Reports*.

[15] Kiernan A. E., Pelling A. L., Leung K. K. H. (2005). Sox2 is required for sensory organ development in the mammalian inner ear. *Nature*.

[16] Neves J., Kamaid A., Alsina B., Giraldez F. (2007). Differential expression of Sox2 and Sox3 in neuronal and sensory progenitors of the developing inner ear of the chick. *Journal of Comparative Neurology*.

[17] Steevens A. R., Sookiasian D. L., Glatzer J. C., Kiernan A. E. (2017). SOX2 is required for inner ear neurogenesis. *Scientific Reports*.

[18] Lavado A., Oliver G. (2007). Prox1 expression patterns in the developing and adult murine brain. *Developmental Dynamics*.

[19] Pistocchi A., Gaudenzi G., Carra S., Bresciani E., del Giacco L., Cotelli F. (2008). Crucial role of zebrafish prox1 in hypothalamic catecholaminergic neurons development. *BMC Developmental Biology*.

[20] Bermingham-McDonogh O., Oesterle E. C., Stone J. S., Hume C. R., Huynh H. M., Hayashi T. (2006). Expression of Prox1 during mouse cochlear development. *Journal of Comparative Neurology*.

[21] Fritzsch B., Dillard M., Lavado A., Harvey N. L., Jahan I. (2010). Canal cristae growth and fiber extension to the outer hair cells of the mouse ear require Prox1 activity. *PLoS One*.

[22] Scheffer D. I., Shen J., Corey D. P., Chen Z. Y. (2015). Gene expression by mouse inner ear hair cells during development. *The Journal of Neuroscience*.

[23] Lawoko-Kerali G., Rivolta M. N., Holley M. (2002). Expression of the transcription factors GATA3 and Pax2 during development of the mammalian inner ear. *Journal of Comparative Neurology*.

[24] Kirjavainen A., Sulg M., Heyd F. (2008). Prox1 interacts with Atoh1 and Gfi1, and regulates cellular differentiation in the inner ear sensory epithelia. *Developmental Biology*.

[25] Stone J. S., Shang J. L., Tomarev S. (2003). Expression of Prox1 defines regions of the avian otocyst that give rise to sensory or neural cells. *Journal of Comparative Neurology*.

[26] Hutson M. R., Lewis J. E., Nguyen-Luu D., Lindberg K. H., Barald K. F. (1999). Expression of Pax2 and patterning of the chick inner ear. *Journal of Neurocytology*.

[27] Rinkwitz-Brandt S., Hans-Henning A., Bober E. (1996). Regionalized expression of *Nkx5-1*, *Nkx5-2*, *Pax2* and *sek* genes during mouse inner ear development. *Hearing Research*.

[28] Li H., Liu H., Corrales C. E., Mutai H., Heller S. (2004). Correlation of Pax-2 expression with cell proliferation in the developing chicken inner ear. *Journal of Neurobiology*.

[29] Favor J., Sandulache R., Neuhauser-Klaus A. (1996). The mouse *Pax2*^1Neu^ mutation is identical to a human *PAX2* mutation in a family with renal-coloboma syndrome and results in developmental defects of the brain, ear, eye, and kidney. *Proceedings of the National Academy of Sciences of the United States of America*.

[30] Dabdoub A., Puligilla C., Jones J. M. (2008). Sox2 signaling in prosensory domain specification and subsequent hair cell differentiation in the developing cochlea. *Proceedings of the National Academy of Sciences of the United States of America*.

[31] Puligilla C., Kelley M. W. (2017). Dual role for Sox2 in specification of sensory competence and regulation of Atoh1 function. *Developmental Neurobiology*.

[32] Warchol M. E., Richardson G. P. (2009). Expression of the Pax2 transcription factor is associated with vestibular phenotype in the avian inner ear. *Developmental Neurobiology*.

[33] Wooltorton J. R. A., Gaboyard S., Hurley K. M. (2007). Developmental changes in two voltage-dependent sodium currents in utricular hair cells. *Journal of Neurophysiology*.

[34] McLean W. J., McLean D. T., Eatock R. A., Edge A. S. B. (2016). Distinct capacity for differentiation to inner ear cell types by progenitor cells of the cochlea and vestibular organs. *Development*.

